# Fingerprints of CD8^+^ T cells on human pre-plasma and memory B cells

**DOI:** 10.1371/journal.pone.0208187

**Published:** 2018-12-12

**Authors:** Ulrike Strittmatter-Keller, Caroline Walter, Celine Rauld, Nicole Egli, Camille Regairaz, Sabine Rabe, Gerhard Zenke, José Carballido, Tamás Schweighoffer

**Affiliations:** Novartis Institutes for Biomedical Research (NIBR), Basel, Switzerland; Institut Cochin, FRANCE

## Abstract

Differentiation of B cells is a stringently controlled multi-step process, which is still incompletely understood. Here we identify and characterize a rare population of human B cells, which surprisingly carry CD8AB on their surface. Existence of such cells was demonstrated both in tonsils and in human apheresis material. Gene expression profiling and real time PCR detected however no CD8A or CD8B message in these cells. Instead, we found that surface CD8 was hijacked from activated CD8+ T cells by a transfer process that required direct cell-to-cell contact. A focused transcriptome analysis at single cell level allowed the dissection of the CD8 positive B cell population. We found that the affected cells are characteristically of the CD27+CD200- phenotype, and consist of two discrete late-stage subpopulations that carry signatures of activated memory B like cells, and early plasmablasts. Thus, there is only a restricted time window in the differentiation process during which B cells can intimately interact with CD8+ T cells. The findings point to a novel link between the T and B arms of the adaptive immune system, and suggest that CD8+ T cells have the capability to directly shape the global antibody repertoire.

## Introduction

Upon antigen encounter, naive B cells undergo a strictly controlled maturation and selection process before they eventually turn into plasma cells with high antibody secretion capabilities. Most of the crucial steps occur in germinal centers (GCs) of secondary lymphoid organs (reviewed in [1,2], where their fate is primarily determined by interactions with two cell types, (a) follicular dendritic cells (FDCs), which serve as antigen reservoir and are the major antigen presenting cells starting the affinity maturation process, and (b) germinal center T cells, that provide cognate help to B cells, mainly via the CD40-CD40L pathway. A third type of cells, CD4+ T follicular helper cells (TFHs) are then required to complete the differentiation of B cells, and to instruct them to leave to GC area [3]. Besides physical cell-to-cell interactions, all these cells also release cytokines that are responsible for maintaining the GC environment, regulate recruitment and release of cells, and shape the response. On the other hand, contribution of other GC associated cells, in particular CD8+ T cells, to B cell differentiation remains largely unmapped.

B cells that successfully complete selection and maturation programs become either antibody secreting plasma blasts / plasma cells, or become memory B cells that guarantee fast responses upon a rechallenge with their cognate antigen. The sequence of developmental steps have been mapped using surface markers and gene expression signatures with increasing resolution, and resulted in a thorough understanding of the discrete stages of cellular development [4]. An important finding that emerged from these studies was that especially memory B cells are more a collection of different subpopulations, rather than a phenotypically and functionally homogenous cell type. Besides the classical memory B cells that are carrying the canonical memory marker CD27, various reports identified a number of non-classical memory-like subsets that often lack CD27, but can be distinguished for example by increased expression of negative signal modulators, such as FCRL4 and FCRL5 [5], or in contrast, by decreased expression of positive regulators like CD21 [6]. Age and ongoing or past infections, may also leave scars behind that additionally complicate the precise classification of late stage B cells [7].

However, the differentiation processes take place primarily in restricted compartments, such as tonsils and lymph nodes, and under normal circumstances, the affected cells can not be observed in the periphery. We analyzed leukapheresis material obtained from normal donors of hematopoietic precursor cells, which offers a unique opportunity to observe rare or hidden immune cell types [8,9]. Donors usually undergo a G-CSF and anti-CXCR4 treatment, which causes redistribution and mobilization of all varieties of lympho-hematopoietic cell types at any stages between precursor and fully mature forms including plasma cells, from the bone marrow, secondary lymphoid organs, and even peripheral tissues [10].

We have consistently observed a small subset of B cells, which surprisingly appeared as phenotypically CD8+ in flow cytometry, both in the leukapheresis material and in tonsils. We found that instead of endogenous mRNA expression and translation within the B cells, the CD8 molecule was obtained in protein form as a result of specific cell-cell interaction between B cells and CD8+ T cells. Transfer of CD8 molecules to B cells was then reproduced and experimentally verified in vitro using cells from normal donors. We used extensive gene and protein expression profiling at the single cell level to characterize those B cells, which are recipients of CD8, and found that B cells can only acquire CD8 at a critical, sensitive stage, when they commit their development towards memory B or plasma cells. Whereas numerous previous studies emphasized the role of CD4+ T cells and TFHs in controlling the fate of B cells in germinal centers and beyond [11], our data point to a contribution of CD8+ T cells in these processes.

## Results

### A population of peripheral B cells carry CD8 on their surface without expressing a CD8 message

**Fig 1:**
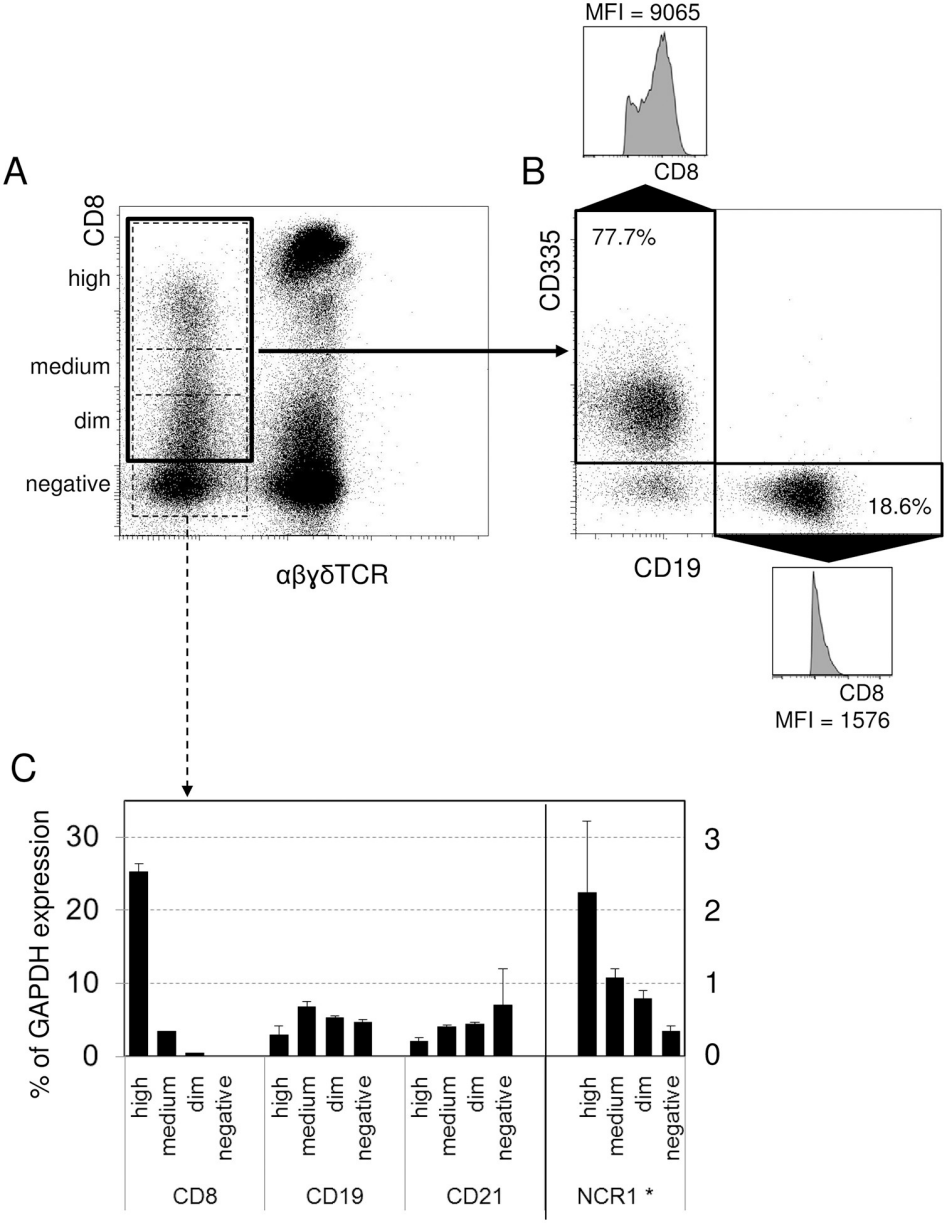
A phenotypically CD8 positive subset of human B cells. Mobilized apheresis material was analyzed with multicolor flow cytometry. The CD8+ TCR negative population was gated (A), and the cells included in the thick black gate resolved using the markers CD335 for NK cells and CD19 for B cells (B). Percentage of both populations is given in the quadrants, and black arrows point to the CD8 expression profiles and MFI values of the boxed CD335+ NK cells and CD19+ B cells, respectively. One representative donor out of four shown. (C) TCR-negative cells were flow sorted into CD8 high, CD8 medium, and CD8 dim populations along with TCR-CD8- cells as negative controls, as indicated by the dashed boxes on panel (A). Expression of genes in the sorted population was then measured by quantitative real-time PCR, and is shown as percentage of the GAPDH housekeeping gene (* for NCR, encoding CD335, the scale on the right hand side applies). One representative donor out of two shown.

We have analyzed apheresis material from healthy human donors who received mobilization treatment with G-CSF, supplemented in some cases by the CXCR4 antagonist Plerixafor. Multiparameter flow cytometry indicated the existence of a sizeable TCRαβɣδ negative (non-T) population that carried variable amounts of CD8 (thick quadratic gate on Fig 1A). The identity and composition of these cells was then surveyed with a panel of mAbs (LegendScreen) that covered most of the known CD specificities. This revealed that the CD8+ non-T cell pool was composed mainly of CD335+ NK cells, but surprisingly also contained a significant proportion of CD19+ B cells (Fig 1B, quadratic gates). The NK cells showed a bimodal distribution of CD8+, with many of them being highly CD8+ (mean fluorescence intensity (MFI) of CD8 signal = 9065). In contrast, the B cell pool was smaller and had a lower average staining intensity (average MFI = 1576), yet still significantly above the level of negative cells.

We then sorted the TCR negative lymphocytes according to their CD8A surface expression levels into four fractions (high, medium, dim, and negatives, as indicated by the dashed boxes in Fig 1A), and determined the mRNA levels of subpopulation-relevant markers by quantitative PCR (Fig 1C). Results indicated that substantial amounts of CD8A message were only present in the phenotypically highest CD8+ cells, with traces in the medium fraction, and lack of message in the CD8dim population. This pattern correlated with the NCR1 message (coding for CD335), suggesting that within the TCR negative cells, NK cells were the sole origins of CD8A message. In contrast, B cells were present in increasing amounts in the fractions that did not produce CD8A messages, as indicated by the CD19 and CD21 mRNA levels. This suggested that B cells were genetically CD8 negative, despite of attaining CD8 positivity in FACS analyses. Nevertheless, only a subset of B cells appeared as CD8+ / dim, while the majority of B cells were still CD8 negative.

**Fig 2:**
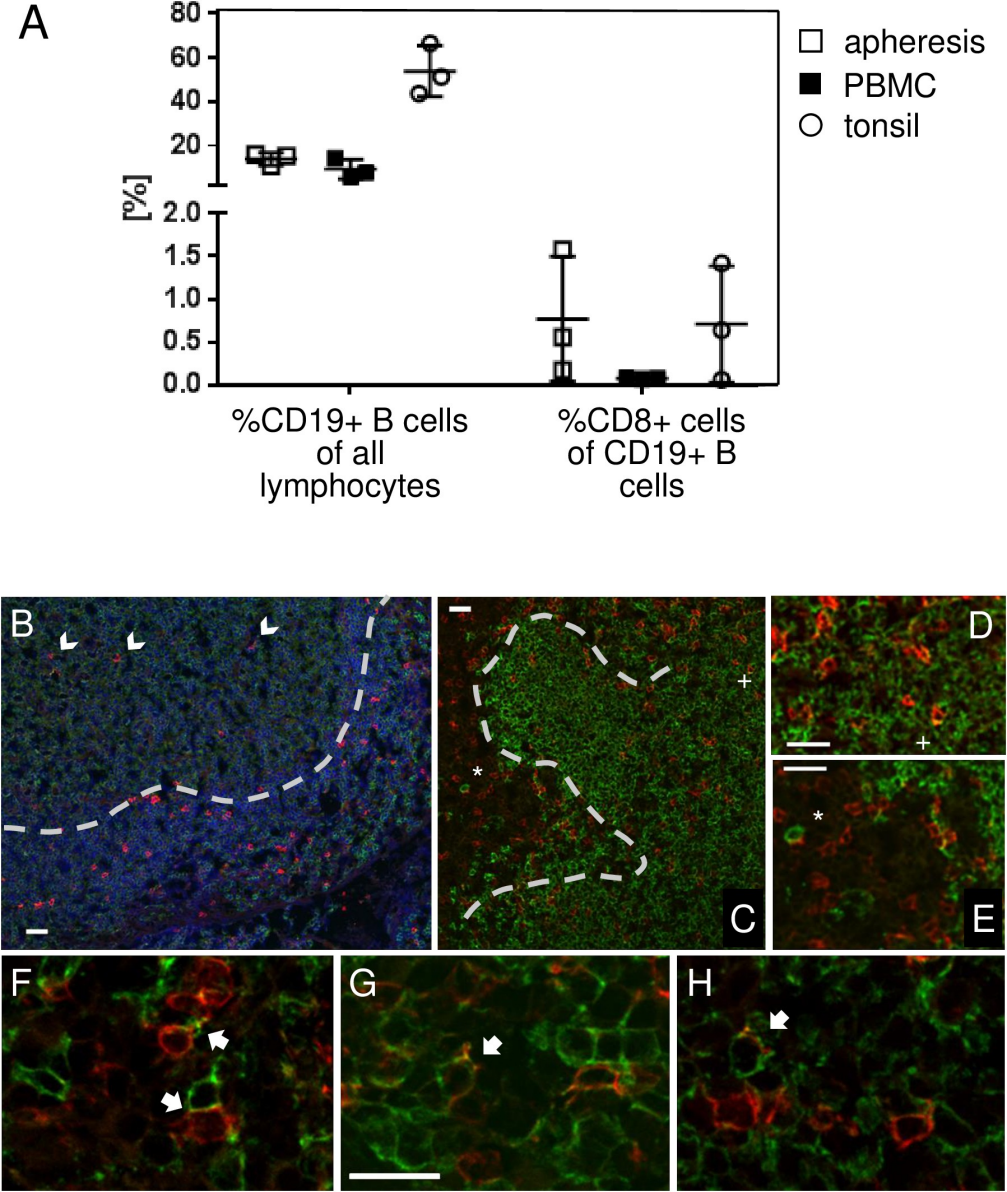
CD8 positive B cells in human tonsil. (A) Subsets were enumerated from apheresis material (open squares), normal peripheral blood (black squares), and from tonsils (open circles) from three donors each. Average and high / low ranges are indicated by lines. (B) Localization of CD8+ T (red) and CD20+ B cells (green) in human tonsils. CD8+ T cells are rare in the germinal centers (GC; arrowheads in panel B), and appear mostly in the mantle zone (MZ; separated by dashed line from the GC). B cells carrying CD8 staining in their membranes were identified in the MZ and in the interfollicular region (panel C, marked with a + and *, respectively; shown at higher magnification on panels D and E). Examples of stable B—CD8+T cell contacts (arrowheads, F), and B cells integrating CD8-carrying patches in their membranes (arrowheads, panels G and H) were identified in the mantle zone. White bars indicate 20um.

TCRαβɣδ- CD8+CD19+ cells were detected in all tested apheresis donors: on the average, 14 ± 2.9% (range: 10.7–16.1%) of all lymphoid elements in the apheresis material were CD19+ B cells, and 0.8 ± 0.7% (0.18–1.58%) of these were CD8+. PBMCs obtained from normal (untreated) blood donors had a somewhat lower content of total B cells (9.45%±4.41%), and these were only rarely CD8+ (0–0.1%). As expected, tonsils had the highest content of B cells (53.8%±11.6%), with a similar frequency of CD8+ B cells as in apheresis material (mean: 0.7%±0.7%, range: 0.1–1.42%) (Fig 2A).

We have also identified CD8+ B cells in situ in human tonsils. In general, CD8+ T cells are rare in the germinal centers (Fig 2B), and appear mostly in the interfollicular and mantle zone (Fig 2B and 2C). B cells carrying CD8 staining in their membranes were localized in these latter regions, where they formed stable B—T cell contacts, acquired CD8, and moved along carrying integrated CD8-containing patches in their membranes (Fig 2D to 2H).

### Phenotypically CD8 positive B cells share characteristics of memory B cells and plasmablasts

**Fig 3:**
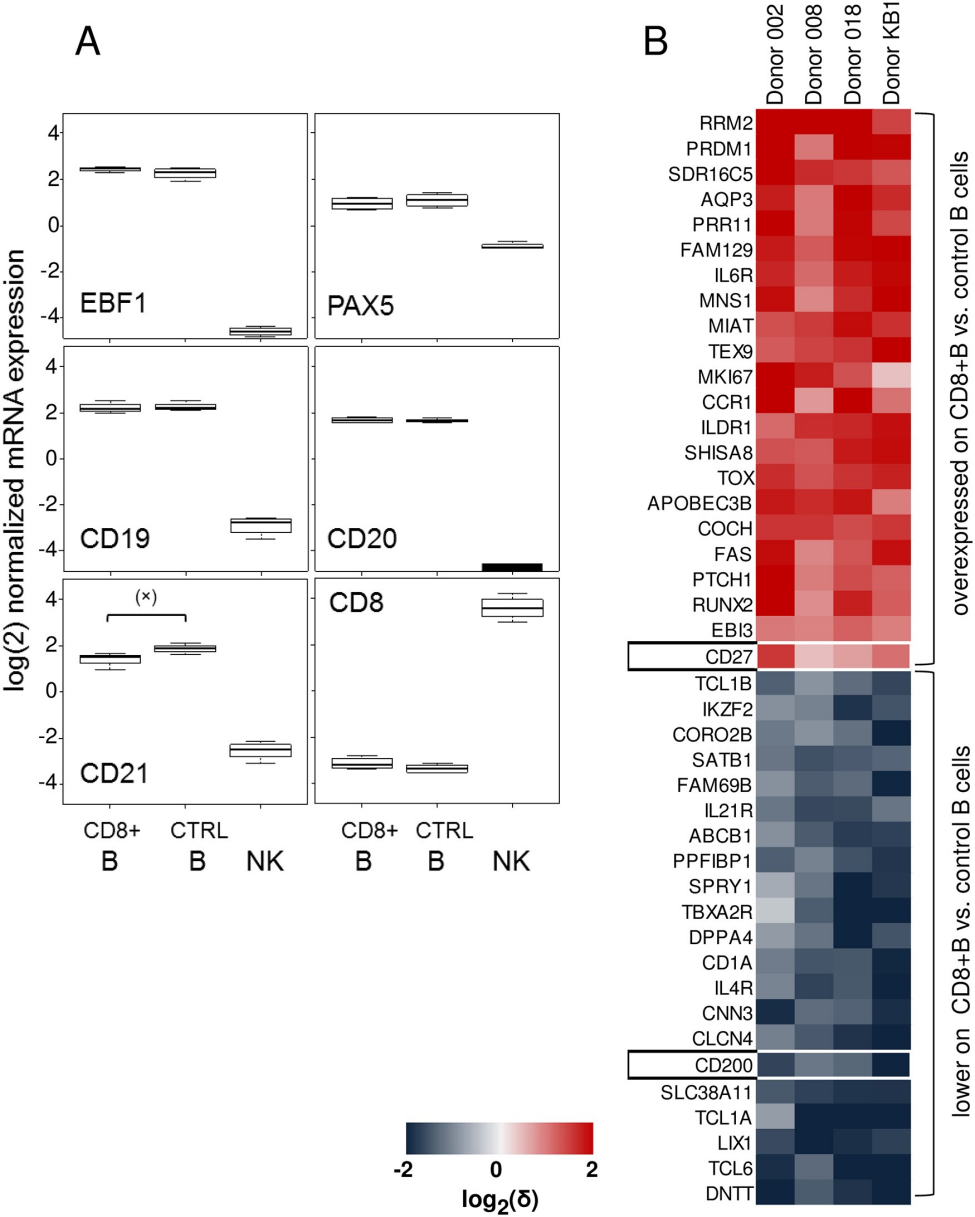
Phenotypically CD8positive B cells belong to a differentiated subpopulation characterized by CD200-CD27++. (A) Comparison of genes expressed by CD8+ B cells, control (CTRL) CD8- B cells, and NK cells. Boxplots represent log(2) normalized data obtained from Affymetrix gene chip measurements of cells sorted from four independent apheresis donors. Differences between the B cell populations vs. NK cells are all highly significant for all genes; (x) indicates a slightly significant (p = 0.048) difference between CD8+ B and control B cells in this dataset. (B) Heatmap of the top upregulated (red) and downregulated (blue) genes on CD8+B cells in comparison to control CD8- B cells. Data represent normalized Affymetrix gene chip measurements of cells sorted from four independent apheresis donors.

To understand whether and how phenotypically CD8+ B cells differ from other B cells, we precision sorted both CD8 positive and negative B cell populations, along with NK cells as controls. Genome-wide expression analysis revealed that the two B cell pools were profoundly similar, and had similar mRNA levels of lineage-defining transcription factors, like EBF1 or PAX5, and of canonical B lineage-specific molecules, like CD19, and CD20. In this class of molecules, only CD21 showed a drop in expression (p = 0.048) in CD8+ B cells (Fig 3A), while changes in many other functionally relevant molecules, like CXCR5, FCRL5, or CD40L, were insignificant (S1 Fig). CD8A expression remained at background levels in both B cell populations, reaffirming the qPCR results obtained from the crudely fractionated cells, whereas as expected, NK cells expressed CD8A at very high levels. Overall, only a small group of genes were differentially expressed on CD8 positive vs. control B cells, whereas NK cells substantially differed from these. The top genes that were up-, or down-regulated on the CD8+ B population compared to conventional B cells are shown as a heat map of individual samples from four independent donors (Fig 3B). Differences between the two pools affect multiple genetic pathways, comprising cell cycle (RRM2, MKI67), cytokine receptors (IL6R up, while IL21R downregulated), but also lineage related, identity determining genes. This was most prominently exemplified by the upregulation of PRDM1/BLIMP1 on CD8+ B cells, which is a key transcription factor for the plasma cell differentiation program [12].

**Fig 4:**
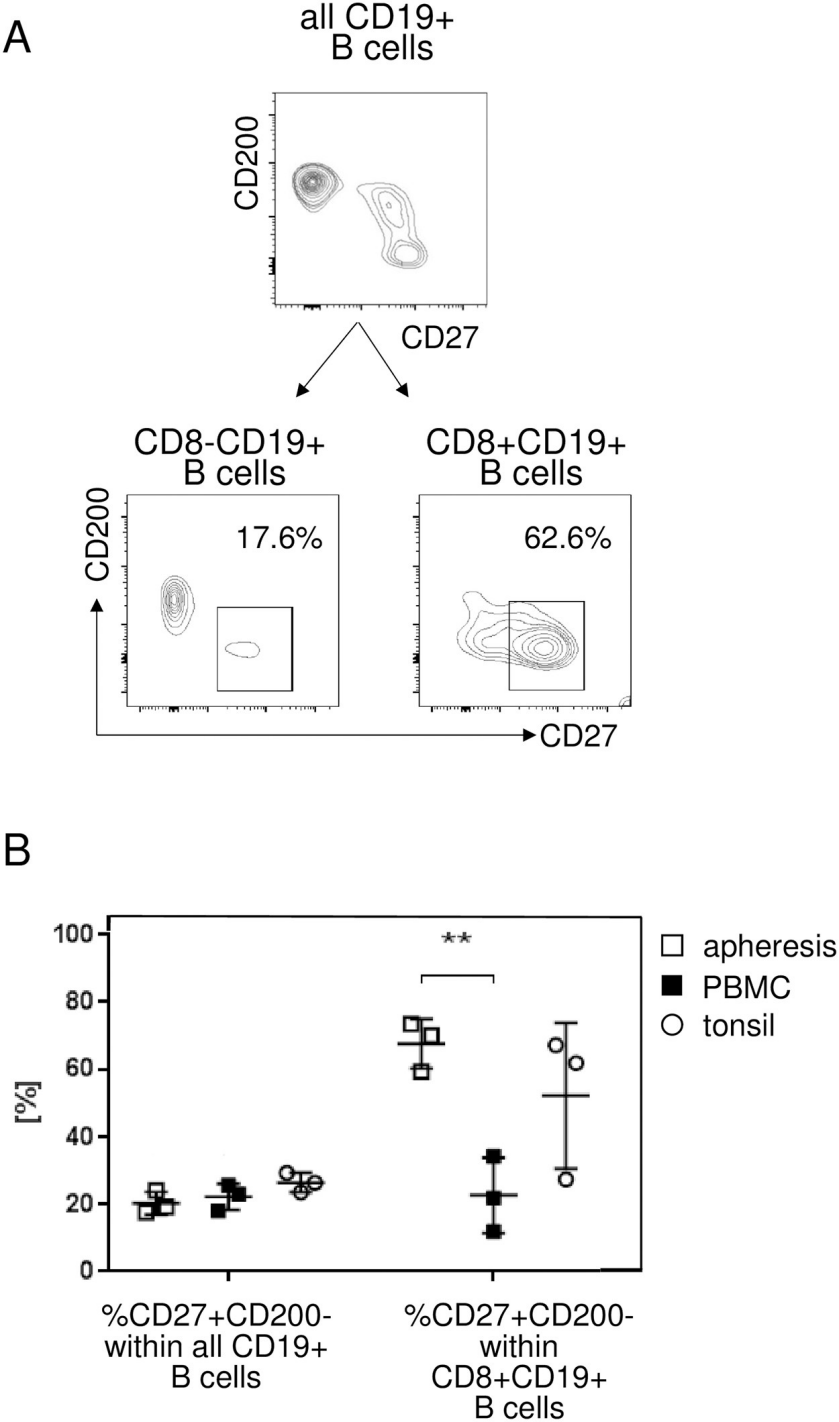
Distribution of B cell subsets in the CD27/CD200 space. (A) In a multicolor FACS analysis, CD19+ B lymphocytes were gated first (top), and these divided then into CD8- (left panel) and CD8+ fractions (right panel). Distribution of these cell subsets as defined by CD27 and CD200 expression are shown as probability plots. One representative apheresis material out of four independent donors tested shown. (B) Proportion of CD27+CD200- cells within CD19+ B cells and the proportion of CD8+CD19+ cells within the CD19+CD27+CD200- pool are shown from apheresis material (open squares), normal peripheral blood (black squares), and from tonsils (open circles). Average and high / low ranges are indicated by lines. (**) indicates p<0.01.

Cell surface proteins from this set were then individually tested in flow cytometry to directly verify subset differences. We found that by using a combination of only two differentially regulated markers, CD27 and CD200 (boxed in Fig 3B), it was possible to dissect the CD19+ B cell pool into preferentially CD8 positive and negative fractions (Fig 4A). CD8 positivity clearly segregated with the CD27+CD200low-negative phenotype. For example, while overall 17.6% of the CD8- B cells in an apheresis donor had a CD27+CD200- phenotype, 62.6% of the CD8+ B cells fell into this gate. The frequency of CD27+CD200- cells was then determined on apheresis, peripheral blood, and tonsil samples obtained from independent donors (Fig 4B). In apheresis material, 20.3±2.9% of total B cells were CD27+CD200-, and both normal PBMC (22.2±3.8%) and tonsils (26.4±2.9%) were in the same range. In contrast, within the CD8+ B cell pool the percentage of CD27+CD200- cells was high in both apheresis (67.6±7.4%) and tonsils (52.2±21.6%), whereas in PBMC (22.7±11%) this percentage was significantly lower. These data likely reflect that CD8+ B cells reside mainly in lymphoid organs, from where they may be mobilized and collected by apheresis, but are otherwise rare in the periphery.

**Fig 5:**
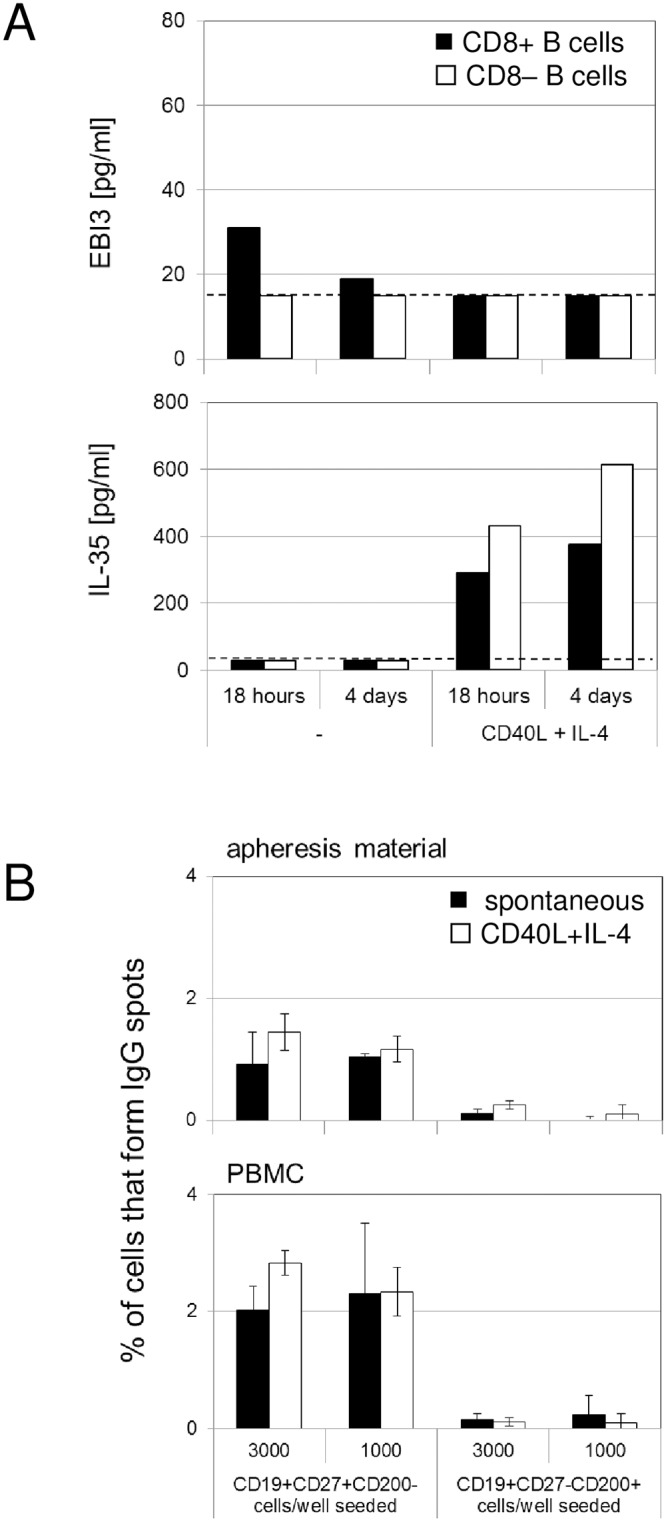
CD8 positive B cells release cytokines and immunoglobulins. (A) Production of the cytokines EBI3 and IL-35 was determined by ELISA from supernatants of freshly sorted CD8+ B cells (black bars) and CD8- control B cells (white bars). Cultures contained 50’000 cells in 200 μl volume. As indicated, samples for both measurements were obtained at 18h and 4 days, without or with CD40+IL-4 stimulation. The dashed line indicates the assay detection limit. (B) Production of IgG was determined by ELISPOT using cells sorted either from apheresis material or from peripheral blood (PBMC). Spots were counted, and are expressed as percentage of input cells (3’000 or 1’000 cells per well, as indicated). Cultures were performed without (black bars) or with CD40L+IL-4 stimulation (white bars).

CD8+ B cells showed also selectively enhanced expression of EBI3, which is a secreted cytokine member of the IL12 superfamily, and of PRDM1, that indicated commitment towards the plasma cell lineage and antibody production. We measured the EBI3 protein levels in sorted, ex vivo cultured cells using ELISA, and found that low, but significant amounts of EBI3 were indeed spontaneously released by CD8+ B cells, but not by CD8- control B cells. EBI3 can heterodimerize with a p35 subunit, and form IL35. Release of IL35 followed the opposite pattern: it was absent in unstimulated cells, but was released after CD40+IL4 stimulation from both populations (Fig 5A). Other related cytokines that are formed by different combinations of the superfamily, such as IL12, IL23, and IL27, were not detectable in any of these samples (not shown). B cells, sorted from either mobilized apheresis material or from normal PBMCs, were then tested in ELISPOT assays to detect ex vivo IgG production. Frequency of IgG producing cells was up to 1.5% in the CD19+CD27+CD200- subpopulation, and thus clearly higher than in CD19+CD27-CD200+ cells (Fig 5B). Production was spontaneous, and additional stimulation with e.g. CD40L + IL-4 did not result in significantly more spots.

Taken together, based on the overall gene expression pattern, the established role of CD27 as a marker of post-germinal center memory B cells, and the immunoglobulin producing capability, we labeled the CD19+CD27+CD200- population collectively as pre-plasma and memory B cells (ppmB).

### B cells can attain CD8 positive phenotype in vitro

**Fig 6:**
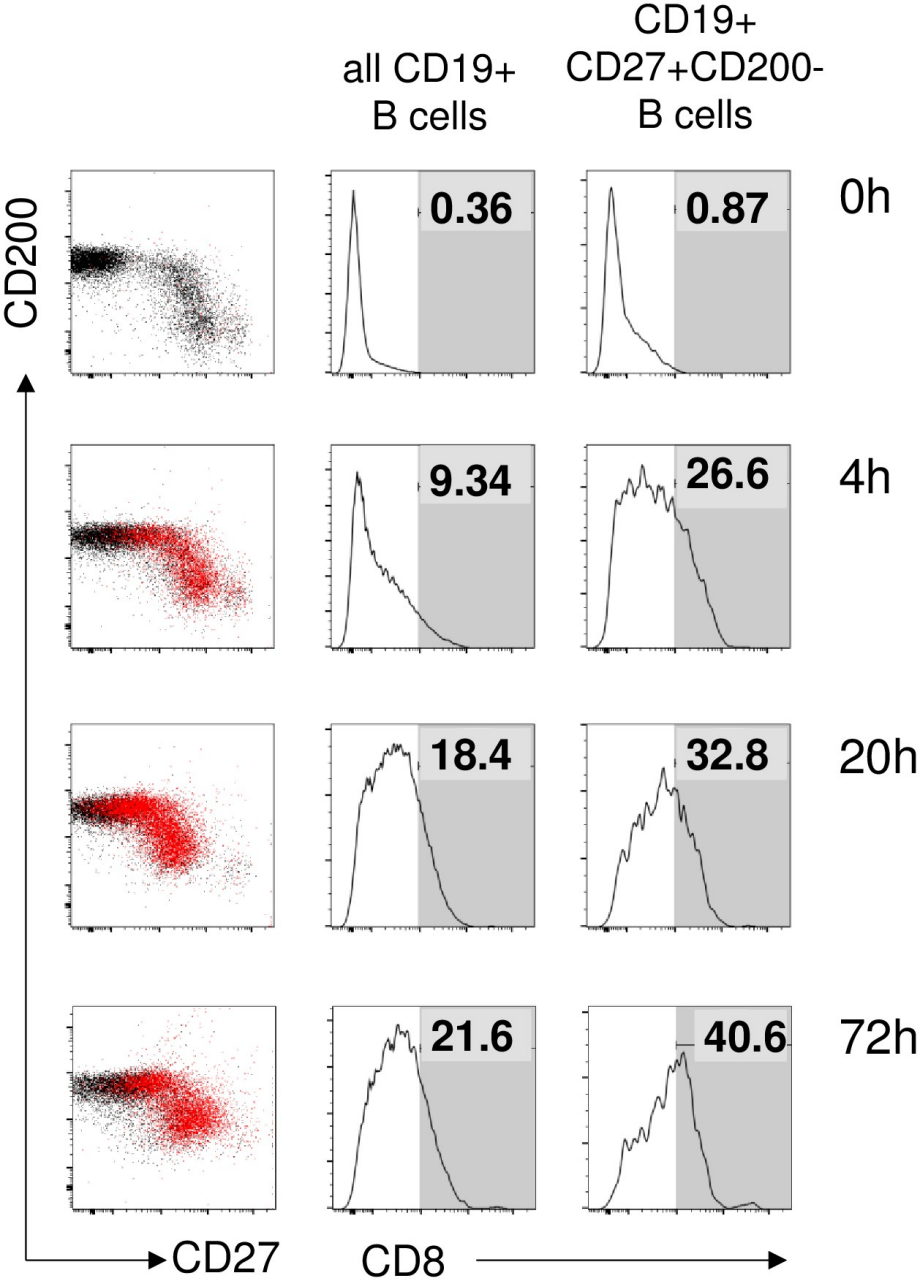
B cells can attain CD8 phenotype in vitro. Conversion of B cells to the CD8+ phenotype was measured by FACS analysis of in vitro PBMC cultures at 0, 4, 20, and 72h. Left column: CD8+CD19+ cells mapped as red dots to the CD27/CD200 space. Middle column: CD8 expression of all CD19+ B lymphocytes, with numbers indicating the percentage of positives (defined as falling into the gate represented by the grey area). Right column: CD8 expression of gated CD19+CD27+CD200- B lymphocytes, with numbers indicating the percentage of positives. Data from one representative donor material out of four tested shown.

The previous data strongly suggested that the presence of CD8 on the surface of B cells was not resulting from endogenous transcription—translation, but was likely acquired in protein form as a result of a transfer process from CD8+ cells. We successfully recapitulated CD8 transfer in vitro in PBMCs, when we included PHA in the cultures to activate T cells (Fig 6). Transfer of CD8A protein to B cells was visible already 4 hours after stimulation, and the signal increased during the next 3 days. B cells were not uniformly good acceptors; instead, also in vitro the CD27+CD200- population was preferentially stained (indicated by red coloring on the left column). While the complementary CD27-CD200+ pool remained mostly negative at the beginning of the cultures, at later timepoints as the interaction between T and B cells proceeded, some of these cells also picked up CD8 (middle and right columns, Fig 6).

**Fig 7:**
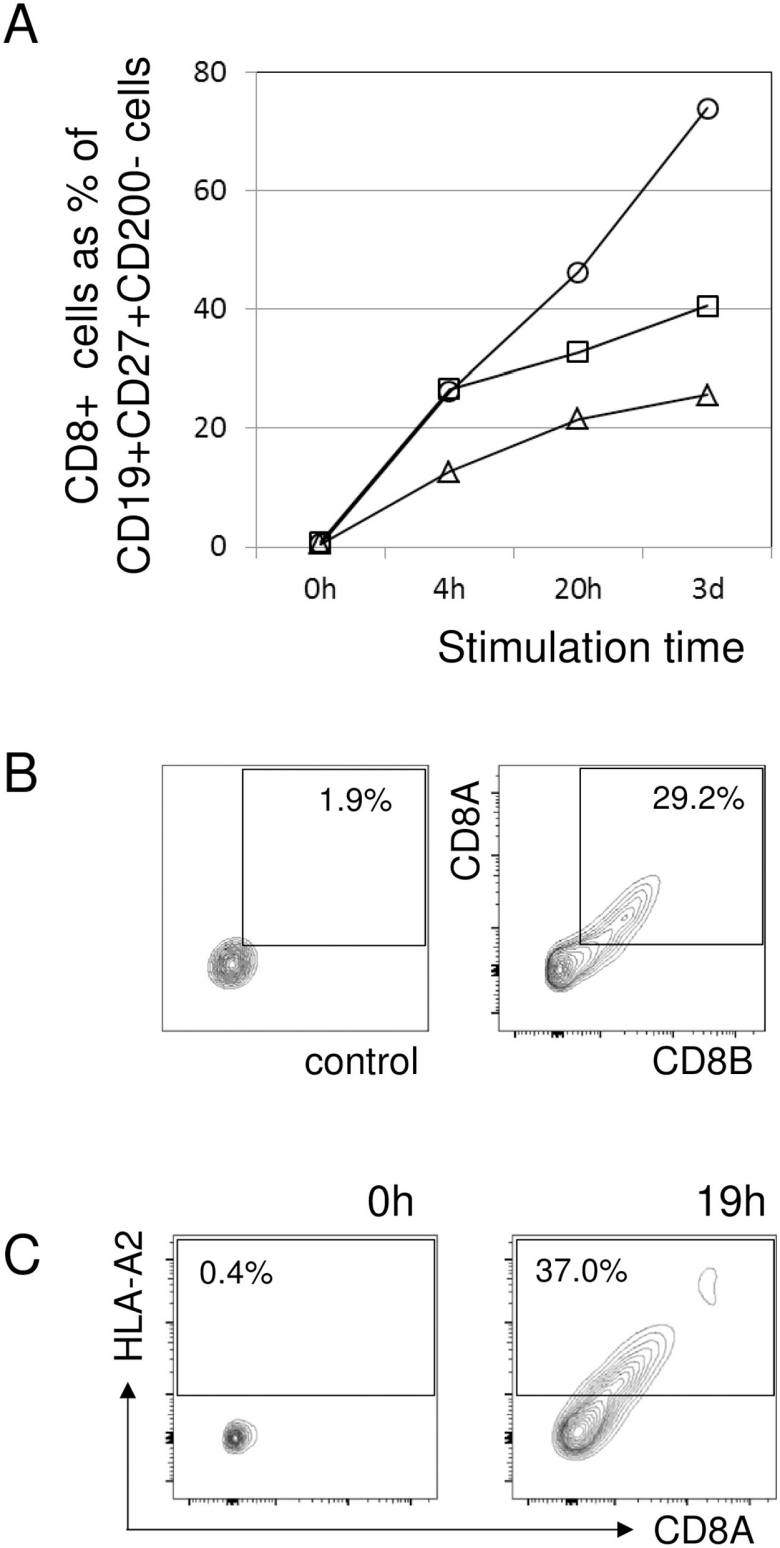
Transfer of molecules to B cells. (A) Percentage of CD8 positive cells within the CD19+CD27+CD200- B lymphocytes is increasing with cultivation time. Each series represents an independent blood (PBMC) donor. (B) Coexpression of CD8A and CD8B on CD19+CD27+CD200- B lymphocytes from a representative apheresis donor (right panel; left panel is the matching control). (C) HLA-A2 and CD8A are coexpressed on CD19+CD27+CD200- B lymphocytes, after CD8-depleted PBMC from an HLA-A2 negative donor were cocultured in vitro for 19h together with positively selected CD8+ T cells from an allogeneic HLA-A2+ donor (right panel; left panel is the expression at 0h / start of the culture).

Similar results were reproducibly obtained with several independent donors, in one case up to 77% of the CD27+CD200- B cells attained CD8 positivity (Fig 7A). CD8+ T cells had to be present in the PBMC during stimulation; when these were removed by magnetic beads, none of the CD19+ B cells became positive for CD8 in flow cytometry (S2A Fig). Also, direct T-B contact was necessary, as supernatants from stimulated cultures, as well as different forms of recombinant soluble CD8 failed to turn B cells CD8 positive (S2B Fig). We also examined possible transfer of other molecules. First of all, together with CD8A also CD8B was transferred, suggesting that the donors were T cells expressing CD8AB heterodimers, and not NK cells carrying CD8AA homodimers (Fig 7B). Also HLA class I molecules were likely transfer candidates because of their high surface copy number. To test this, CD8+ T cells isolated from an HLA-A2+ donor were co-cultivated with PBMC obtained from an HLA-A2 negative donor that was depleted from CD8+ T cells. In this experimental setting, some T cells could experience allogeneic cross-reactive stimulation, which already resulted in a minimal scattered transfer of molecules. With additional PHA stimulation, along with CD8 also HLA-A2 became increasingly detectable especially on CD19+CD27+CD200- cells; after 19h stimulation up to 37% of CD19+CD27+CD200- cells became HLA-A2 positive (Fig 7C).

### Single cell expression analysis identifies signatures of B cells responsive to CD8+ T cell interactions

**Fig 8:**
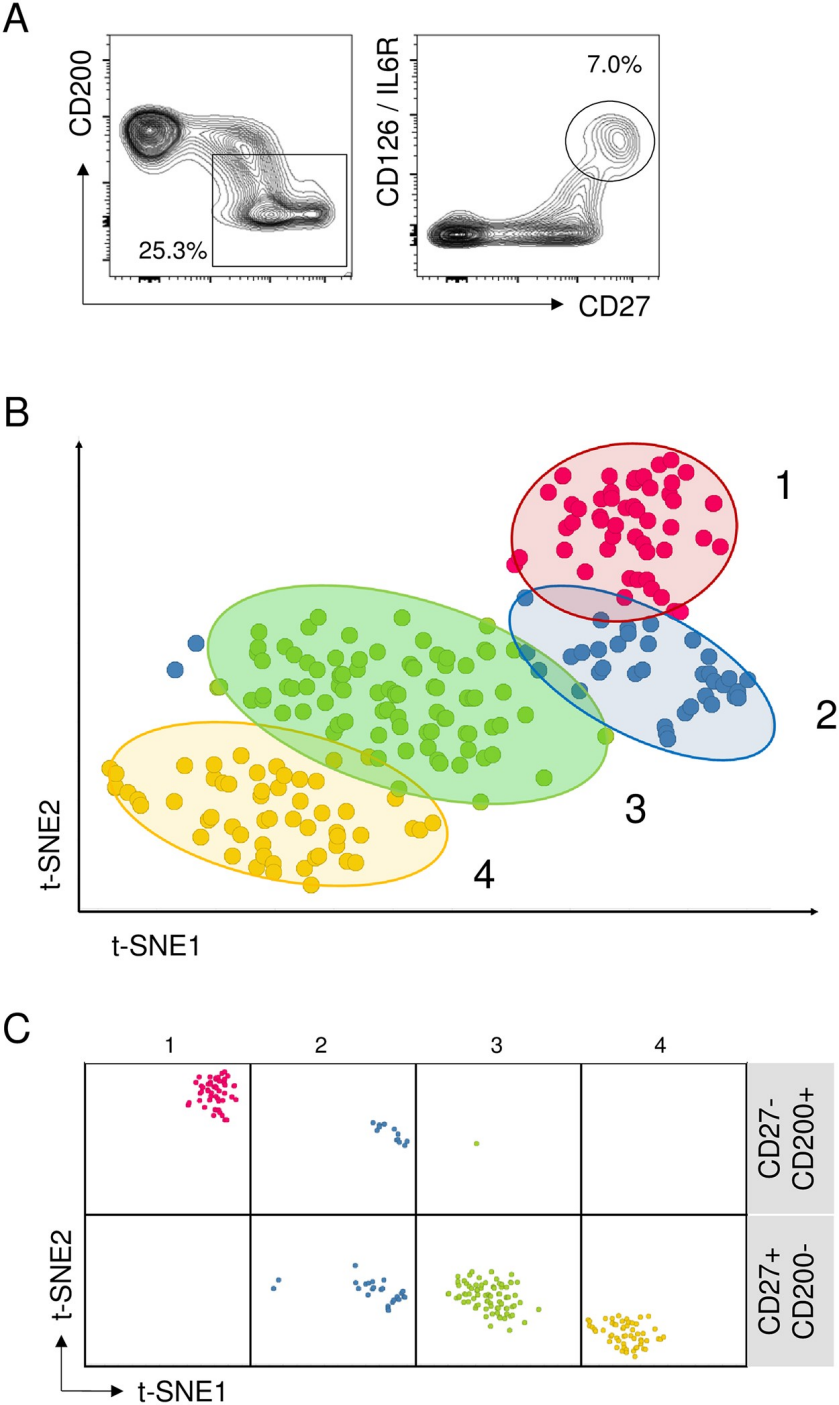
Single cell dissection reveals subsets within the potentially CD8 acceptor B cell pool. (A) IL6R (CD126) is expressed on a small subset of CD19+ cells (right panel), that is only a fraction of the corresponding total CD19+CD27+CD200- pool (left panel). Numbers indicate the percentage of gated cells within all B lymphocytes. (B) Two-dimensional t-SNE analysis of all single cell events (n = 231) pooled from two apheresis and two PBMC donors; each symbol represents an individual cell. Subsets are numbered and color coded according to their assignment by R-PhenoGraph. Ellipses represent the 95% confidence interval for each color matched population. (C) The single cell–based subsets resolved according to their phenotypic (FACSort) origin, using the same t-SNE and R-PhenoGraph annotation as in (B).

Despite of their homogeneity in CD8 uptake, some findings indicated that CD27+CD200- B cells still could represent a moderately complex collection that is heterogeneous in marker expression profiles and functional properties. For example IL6R, which according to the gene expression pattern was upregulated globally on CD8+ B cells, was only detected on less than one-third (7% vs. 25.3%) of these cells (Fig 8A). The low frequency of immunoglobulin producing cells, and the EBI3 release pattern also indicated the possibility that CD8+ B cells contain functionally divergent cells.

Therefore we addressed population heterogeneity at the single cell level. CD19+ CD27+CD200- B cells, along with matched CD19+CD27-CD200+ control B cells, were sorted form various donors, and applied to the C1 microfluidic single cell device [13]. Single cell occupancy of the chambers was verified, and positions of control and test cells were also recorded. Cells were converted individually into cDNA pools, and expression of a panel of genes encompassing both generic B cell markers, as well as genes that were showing expression differences in our previous gene array experiment (Fig 3B), was determined by quantitative real time PCR. qPCR results were expressed as log10 of expression relative to housekeeping genes, whereas non-detects [14] were assigned the value (-5). Data were processed for dimension reduction using the t-SNE method, and thereafter cells were assigned to subsets by the nonbiased population detection algorithm PhenoGraph [15,16]. Experiments from two apheresis and two PBMC samples revealed a consistent pattern, and identified four subsets (Fig 8B). As t-SNE is based on the distance of similarity between the individual gene expression sets, relative localization of the populations may also reflect cellular development stages [17], which we then marked with 1 > 2 > 3 > 4 and a color code. Each donor itself also retained both spread and relative positioning of these subsets, even when the amounts of cells varied within the subsets (S3 Fig).

The distribution of the identified cell subsets was also consistent with the input FACS parameters. Subset_1, identical to the control B cells, and characterized by expression of ABCB1 and TCL1A, was exclusively containing CD27-CD200+ cells. In contrast, CD27+CD200- B cells were dissected into two main populations (subset_3 and _4). A transient type (subset_2) appeared in both sorted pools (Fig 8C).

**Fig 9:**
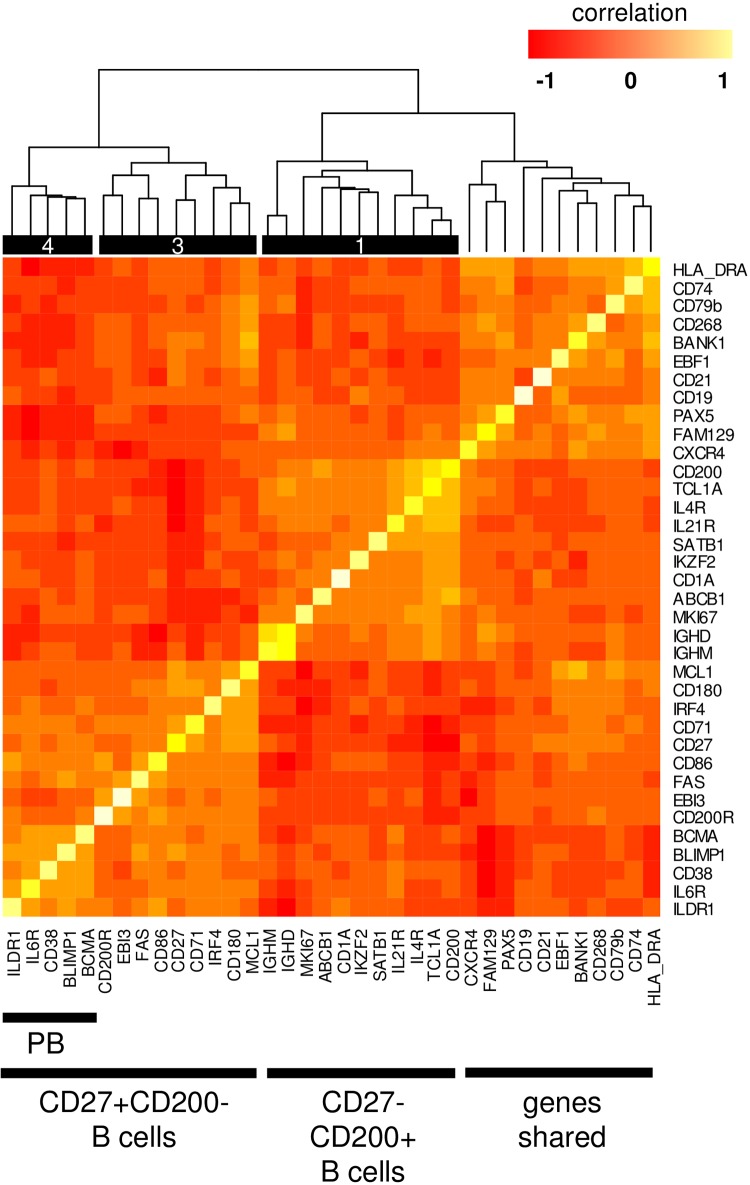
Hierarchically clustered correlation of gene expression from the pooled single cell data. The dataset contained all observations derived from all cells from two apheresis and two PBMC samples with the exception of cells that were negative of every sorting marker.

Expression levels of genes on individual cells, and a hierarchically clustered correlation heat map (S4 Fig) enabled to establish signatures for the subsets and to assign them a more precise identity (Fig 9). CD27, the classic memory B cell marker was associated with subset_3, coclustering with other established markers such as CD71 and IRF4. A discrete signature characterizing subset_4 was formed by genes co-clustering with PRDM1, including CD38, BCMA, and IL6R, which are all known to be induced during terminal differentiation. The molecule that showed the closest correlation with PRDM1 in our set was ILDR1, which can thus serve as a potential novel surface marker for this subset.

**Fig 10:**
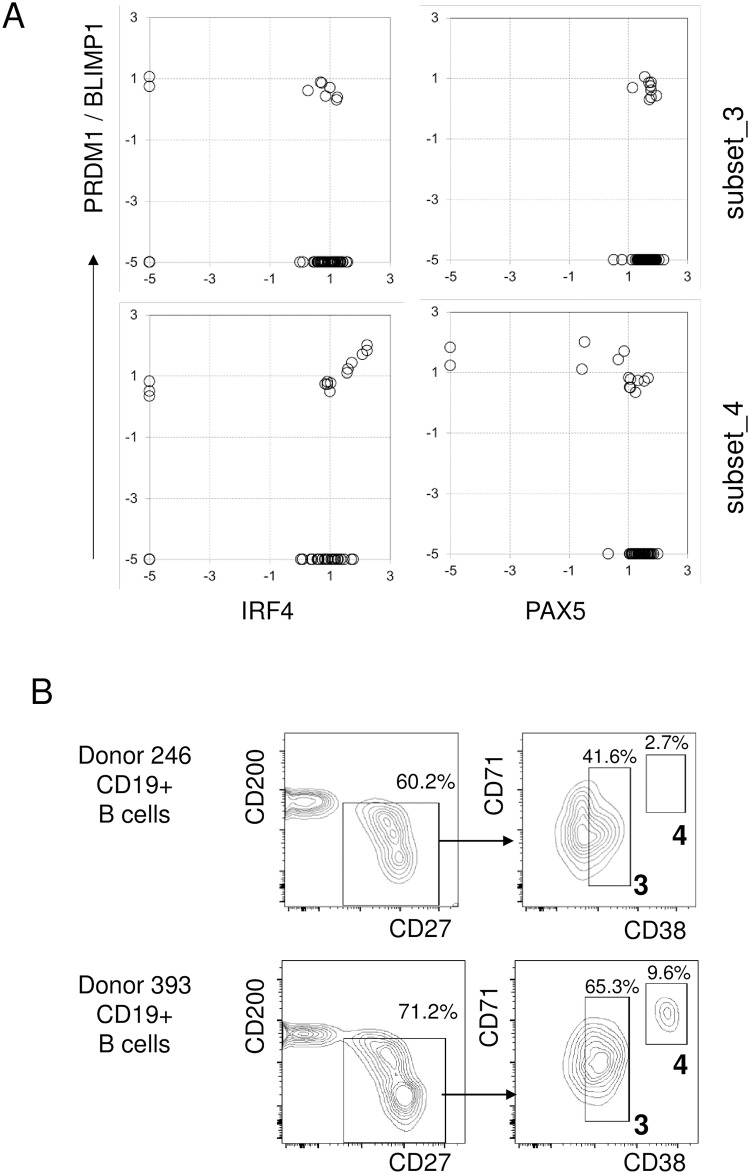
Expression of signature genes. (A) Expression of PRDM1/BLIMP (y axis, log(2) expression) vs. signature genes (x axis, log(2) expression) in subset_3 (upper row) and subset_4 cells (lower row). Each symbol represents an individual cell. (B) PBMC from a selected donor were analyzed in multicolor cytometry and sequentially gated for CD19+ B cells shown in the CD27/CD200 space (left panel); gated cells were then further resolved using CD38 and CD71 staining (right panel). Resolved subsets are boxed and numbered to match the R-PhenoGraph designation.

Key lineage determining markers were compared pairwise on subset_3 and subset_4 cells (Fig 10A). We found that both IRF4 and PAX5 were in general expressed at high levels on most cells, with a few exceptions even in cells where PRDM1 was already turned on. Loss of PAX5, which is an expected consequence of PRDM1 expression during terminal B cell differentiation, was seen only in a few subset_4 cells (Fig 10A lower right panel). Taken together, these data argued that the CD8+ B pool was composed of two major cell types: subset_3, belonging to the post-germinal center memory B cell collection; and subset_4, displaying properties of early plasmablasts with some additional microheterogeneity as revealed by the PRDM1 status.

Based on these gene expression data, we have expanded our flow cytometry panel by antibodies against CD38 and CD71 [18] to examine whether subset_3/4 cells would be detectable in the peripheral blood PBMCs from normal donors, despite of their expected rarity. We identified one sample with an elevated percentage of subset_3 cells (Fig 10B, upper row), and another sample where both subset_3 and subset_4 cells were present in unusually high amounts (Fig 10B, lower row). These findings indicate that such cells can occasionally, under rare circumstances appear in elevated numbers in the peripheral blood, even without the need for pharmacological mobilization.

## Discussion

Lymphoid cells can be reliably assigned to their core lineages based on the expression of specific markers. It was thus rather surprising that we have repeatedly detected CD8 molecules on the surface of a small subset of B cells in GCSF mobilized apheresis material: so far in every examined apheresis sample up to 2% of the B cells were CD8 positive.

Regulation of CD8 expression is directed by a circuit of transcription factors and genome organizers which are understood in great detail [19]. Many of the required molecules are absent in B cells, which per se precludes stable expression of CD8 messenger RNA by B cells; therefore the current consensus is that B cells do not express CD8. As an exception, aberrant CD8 expression was found in a retrospective study in less than 2% of B cell lymphoma cases [20].

We confirmed lack of mRNA expression using quantitative PCR and gene chip analysis in purified, phenotypically CD8 positive B cells, and thus concluded that the appearance of surface CD8 molecules must be due to other mechanisms. Capture of protein molecules, or their exchange between immune cells has been repeatedly observed both in vivo and in vitro, and is likely happening more frequently than anticipated [21]. T cells, NK cells, or macrophages were repeatedly shown to act as acceptors that were decorated with foreign proteins. These cells are capable to acquire a structurally wide variety of molecules, such as MHC/HLA, or costimulatory and signaling molecules with functional impact on the recipients [22–26]. In contrast, B cells were rarely seen as acceptors: while they can apparently routinely acquire soluble antigens by forming synapses [27], only one group reported in vitro transfer of T cell derived molecules to B cells in vitro [28]. Our findings are thus the first precedent of natural, in vivo transfer of cell membrane molecules onto human B cells.

There are multiple ways B cells would be able to acquire CD8. CD8 is also expressed as an alternately spliced soluble variant [29,30], and its extracellular part can be shed by enzymes, so if B cells would express a dedicated receptor, they could easily bind these molecules. Also CD8 could be transferred as a component of membrane derived microvesicles shed by T or NK cells [31]. In our experiments, incubation of B cells with different recombinant soluble sCD8 molecules, or with complete supernatants of resting or stimulated cells did not elicit CD8 expression. We thus concluded that CD8 positivity of B cells had to be a result of an intercellular molecular transfer process that required direct cell-cell contact [21,32–34]. The fact that overall CD8 MFI values on B cells remained significantly lower than on cells expressing endogenous CD8, was also consistent with a transfer mechanism. Using cocultures of B cells and CD8+ T cells from peripheral blood we reproduced the transfer in vitro. As both the CD8A and CD8B chains of the heterodimer were transferred, these must have originated mainly from T cells, and not from CD8AA homodimer expressing NK cells. The transfer event is not restricted to CD8, but can involve other molecules which are colocalized in the transferred membrane patches, as exemplified by the transfer of HLA molecules. Taken together, we conclude that appearance of the CD8 molecules is a result of protein transfer, and as such, is a remnant of an intimate interaction between CD8+ T and B cells.

We sought to understand the differences between CD8 decorated acceptor B cells, and unmodified B cells by analyzing their gene expression profile. We found that by using only two markers, CD27 (upregulated) and CD200 (downregulated), the CD8+ B cells could be clearly separated from other B cells. CD27 is a marker that is robustly upregulated on post-germinal center memory and late-stage circulating cells [4]. The role of CD200 in normal B cell differentiation has not been characterized, but CD200 is being utilized as a marker with prognostic significance for staging of B cell leukemias and other malignancies [35]. In particular, loss of CD200 was observed in mantle cell lymphomas, whose normal counterparts are early GC founder cells [36], suggesting that lack of CD200 may characterize GC and post-GC B cells. Recent data suggest that lower expression of CD21 is characteristic for B cells that recently left the germinal center, and are either activated memory-like B cells [6,37], or are primed for terminal differentiation towards plasma cells [38]. By absolute numbers CD8+ B cells were rare: the CD27+CD200- population comprised about 20% of total B cells, (equivalent to ~40% of the CD27+ global memory B pool), and even in the best apheresis donor only less than 2% carried detectable CD8. These data strongly argued that only B cells that are at a specific stage of their development, and which possess appropriate surface molecules for intercellular interactions, are suitable acceptors of CD8.

We expected a better understanding of the CD8-receptive B cells by analyzing their transcriptional profile individually at the single cell level [39]. Data analysis detected two major discrete and relevant populations in all examined donors. Subset_3, expressing CD27, CD71, and CD86, are clearly GC-to-post-GC memory cells [3,4]. In addition, both MCL1 and CD95/FAS were upregulated in these cells. These two molecules are key regulators of cell survival with antagonistic roles; while MCL1 is a prominent anti-apoptotic molecule in GC / post-GC activated B cells [40], FAS expressing B cells were shown to be more prone to apoptosis, have reduced lifespan and impaired functionality [4,41]. Closest to FAS clustered EBI3, whose production was verified in ELISA. Subset_4, that was positioned most distal to circulating control (naive) cells, was distinguished by the expression of PRDM1, CD38, IL6R, BCMA, and ILDR1. Collectively these genes clearly articulate that subset_4 cells are at a late stage of the B cell differentiation, belonging to the plasma blast / plasma cell group [42]. While according to canonical models [43,44] PRDM1 and PAX5 are mutually exclusive, we have identified several cells within the subset_4 which expressed both genes, together with high levels of IRF4 [45]. These findings suggested that we have observed B cells, which were just at the brink of commitment, and would represent pre-plasmablasts. Within the signature, ILDR1 was the molecule whose expression correlated best with PRDM1. ILDR1 (angulin-2) is a cell surface immunoglobulin superfamily member molecule that mediates adhesive interactions, associated with tricellular tight junctions in the cochlea, and plays a role in water homeostasis in the kidneys [46,47]. While ILDR1 expression was detected in various cell lines, including the U266 myeloma line [48], expression in primary cells and hematopoietic malignancies has not been surveyed. ILDR1 may be thus not only useful as a novel marker for early plasma cells, but may play a functional role in B cell differentiation, perhaps also as an interaction partner that contributes to trogocytosis of CD8.

The signature data support a model where B cells would have a relatively restricted timeframe to interact with CD8+ T cells: after B cells achieve a certain degree of maturation, and around their final commitment towards memory B or plasma cells. This timing suggests that the interaction must take place within or close to germinal centers or GC-like structures. CD8+ T cells that express CXCR5 can migrate into B-cell follicles, and modulate the outcome of antiviral responses [49]. CD8+ B cells also (insignificantly) decreased CXCR5 levels, suggesting that they may move in the opposite direction, disfavoring transfer of CD8 onto B cells inside the GC. While our immunofluorescence stainings of human tonsils detected GC-localized CD8+ T cells, CD8+ B cells were found in the mantle zone and to the interfollicular region. Together these findings suggest that affected memory B cells and plasmablasts migrate outside of the follicles, where they establish close contacts to T cells. It remains to be clarified if this is a transient move, followed by a re-entry into the GC, or perhaps part of the export process shown for plasmablasts [1].

Currently we can only speculate on the functional consequences of a direct CD8+T—B contact. CD8+ T cells, like TFHs, may modify the local cytokine milieu [3,11]. Alternatively, they may act as it is anticipated from a killer cell, and eliminate interacting B cells. Killing of autoreactive plasmablasts by CD8+ T cells would also explain why CD8+ B cells are so scarce in normal PBMC, and become more visible only under special circumstances, like in apheresis material. Failure of such a terminal control may then result in immune escape, potentially leading to autoimmune diseases, monoclonal gammopathies, eventually even to multiple myeloma [50]

In summary, we found that a discrete population of human B cells, which are within a tight window of their late-stage differentiation, carry CD8 on their surface. These CD8 molecules represent a fingerprint of recent interactions, and indicate that affected B cells were in close contact by CD8+ T cells. We propose that the B—CD8+ T cross-talk may function as a gating event that influences terminal B cell differentiation, and shapes the serological immune response, ultimately with possible relevance to pathogen defense and autoimmune diseases.

## Materials and methods

### Cells

Mobilized apheresis material from healthy volunteers, and tonsils from clinical interventions were provided under informed consent (protocol TRI0143) in accordance with the Swiss Human Research Act (HRA; January 2014) and with approval of the responsible ethic committee (Ethikkommission Nordwest- und Zentralschweiz). Samples were either collected by University Hospital Basel with delivery times <2h at ambient temperature; or were obtained from KeyBiologics (Memphis, TN) with 39h delivery time at 4°C in cryopreservation medium. No personal data of donors except for age, sex, and final clinical CD34+ counts were reported. Blood from healthy volunteers was provided under written informed consent and collected through the Novartis Tissue Donor Program (TRI0128), in accordance with the Swiss HRA, and approval of the NIBR Bioethics Advisory Group as Institutional Review Board and responsible ethic committee. Sample life-cycle management and record keeping, including planning, acquisition, collection, storage, release for use, anonymization, and destruction of all human samples, was performed in compliance with the applicable law.

For measuring transfer of CD8, PBMC were prepared by density centrifugation. Cultures were set up in 24well plates at 2x10^6^ PBMC per well in RPMI1640 medium containing 10% FBS and antibiotics, with 1μg/ml PHA-L (Sigma) as stimulus. Where indicated, CD8+ T cells were isolated from PBMCs by immunomagnetic separation (Stem Cell Technologies), according to the manufacturer’s protocol using an EasySep magnet. After stimulation, cells were stained and fixed with paraformaldehyde, and samples were analyzed by an LSR Fortessa equipped with four lasers (Becton Dickinson).

### Antibodies, Elispot, tissue immunfuorescence

Antibodies used were: TCRgd (B1), CD8A (SK1), CD19 (SJ25C1), CD200 (MRC OX-104), CD27 (M-T271), CD126 (IL6R alpha; UV4), HLA-A2 (BB7.2), CD34 (clone 581), CD38 (HIT2), all from Becton Dickinson; TCRab (LZU902, Novartis); CD8B (SIDI8BEE) from eBioscience; CD335 (9E2), CD71 (CYG4) and the LegendScreen antibody set from BioLegend. ELISA kits for measuring human EBI3 and IL-27 were from LifeSpan BioSciences.

The Elispot assay kit for human IgG was from mabtech AB, and the assay was done according to manufacturer instructions. Briefly, filter plates (PVDF membrane, Millipore) were coated overnight with anti-human IgG antibodies at 4°C. Plates were washed with PBS and blocked for at least 30 minutes at room temperature. Blocking medium was removed, and sorted cells were added at 3’000 or 1’000 cells per well in 100 μl culture medium, followed by 100 μl of medium alone or medium with stimulants (recombinant CD40L (Enzo) + recombinant human IL-4 (R&D Systems)). Cells were incubated overnight, then washed with PBS and incubated with biotinylated anti IgG antibodies. Spots were developed using streptavidin-HRP (diluted 1:800), and 3,3′,5,5′-Tetramethylbenzidin (TMB) substrate (all mabtech AB).

For histological localization of CD8a and CD20 expressing cells, frozen sections were prepared from human palatine tonsils embedded in OTC. Sections were blocked, incubated with mouse-anti-human CD20 (clone L26, Dako M0755) and with rabbit-anti-human CD8a monoclonal antibody (D8A8Y, Cell Signaling Technologies 85336), and then with donkey-anti-mouse IgG Alexa488 and donkey-anti-rabbit IgG Alexa568 (both Abcam) secondary antibodies. Cell nuclei were counterstained with DAPI, and slides mounted in ProLong Gold antifade reagent (Invitrogen). Images were collected using an Olympus FV3000A confocal microscope, and processed by Olympus FV31S-SW Fluoview.

### Gene expression and data analysis

Target cell populations were sorted on a FACSAria (Becton Dickinson); post-sort purity was >97%. mRNA expression was determined using Affymetrix HG-U133plus2 chips, and data sets analyzed using GeneSpring, R, and Spotfire. Single cell mRNA expression patterns were determined using the C_1_ Single-Cell Auto Prep System coupled with the Biomard HD instrument and HX IFC controller, all from Fluidigm. Prior injecting cells into the system, CD19+CD27+CD200- cells and CD19+CD27-CD200+ control B cells were sorted based on surface marker expression and live/dead exclusion staining. Control B cells were then stained with Vybrant Dye Cycle Green (Life Technologies #V35004), and then admixed to unstained B cells at a ratio of 1:4. Final concentration was adjusted to 4x10^5^ cells/ml. The Targeted Gene Expression TaqMan Protocol provided by Fluidigm was followed, using a single cell-to-CT kit (Ambion by Life Technologies #4458237), and modules from C_1_ reagent kit (Fluidigm #100–5319), C_1_ Integrated Fluidic Circuit (IFC) for PreAmp (size 5–10μm; Fluidigm #100–5757). All 96 pre-amplified cDNA products were harvested, diluted, and prepared for real time PCR with 42 TaqMan gene assays of interest following the 96.96_GE_TaqMan-Fast protocol using a 96.96 Dynamic Array IFC (Fluidigm #BMK-M-96.96). Results were analyzed with software provided by Fluidigm, and exported for normalization (as percentage of EF1ɑ and GAPDH housekeeping genes). A log10 transformation was applied, and only data that originated from single living cells were kept for further analysis. The cytofkit Bioconductor package implemented in R was used to calculate t-SNE parameters and to define cell clusters [16]. Results were visualized with Spotfire (TIBCO); statistics, boxplots, clustering, and heatmaps were prepared in R.

## Supporting information

S1 Fig Other functional markers expressed by CD8 positive and negative B cells. Comparison of genes expressed by CD8+ B cells, control (CTRL) CD8- B cells, and NK cells. Boxplots represent log(2) normalized data obtained from Affymetrix gene chip measurements of cells sorted from five independent apheresis donors.
(TIF)

S2 Fig Conditions tested for CD8 transfer. (A) PBMC and CD8 depleted PBMC were cultured with or without PHA-L stimulation (1 μg/ml) for the indicated time. (B) Summary of other transfer tests. Supernatants were obtained from various cell culture preparations, and incubated with purified B cells. Results are expressed as percent of CD8+ cells within the CD19+CD27+CD200- population.
(TIF)

S3 Fig tSNE by donor. Two-dimensional t-SNE plot of single cells obtained from two PBMC (upper panels) and two apheresis (lower panels) donors.
(TIF)

S4 Fig Hierarchical clustering of subsets based on their gene expression pattern. Comparison of control B cells and subsets 3 and 4, based on the converted relative log expression of all tested genes.
(TIF)
