# Supplementary figures and images for: Fingerprints of CD8+ T cells on human pre-plasma and memory B cells
Source: PLoS One. 2018 Dec 12;13(12):e0208187. doi: 10.1371/journal.pone.0208187 (PMC6291140; doi:10.1371/journal.pone.0208187)

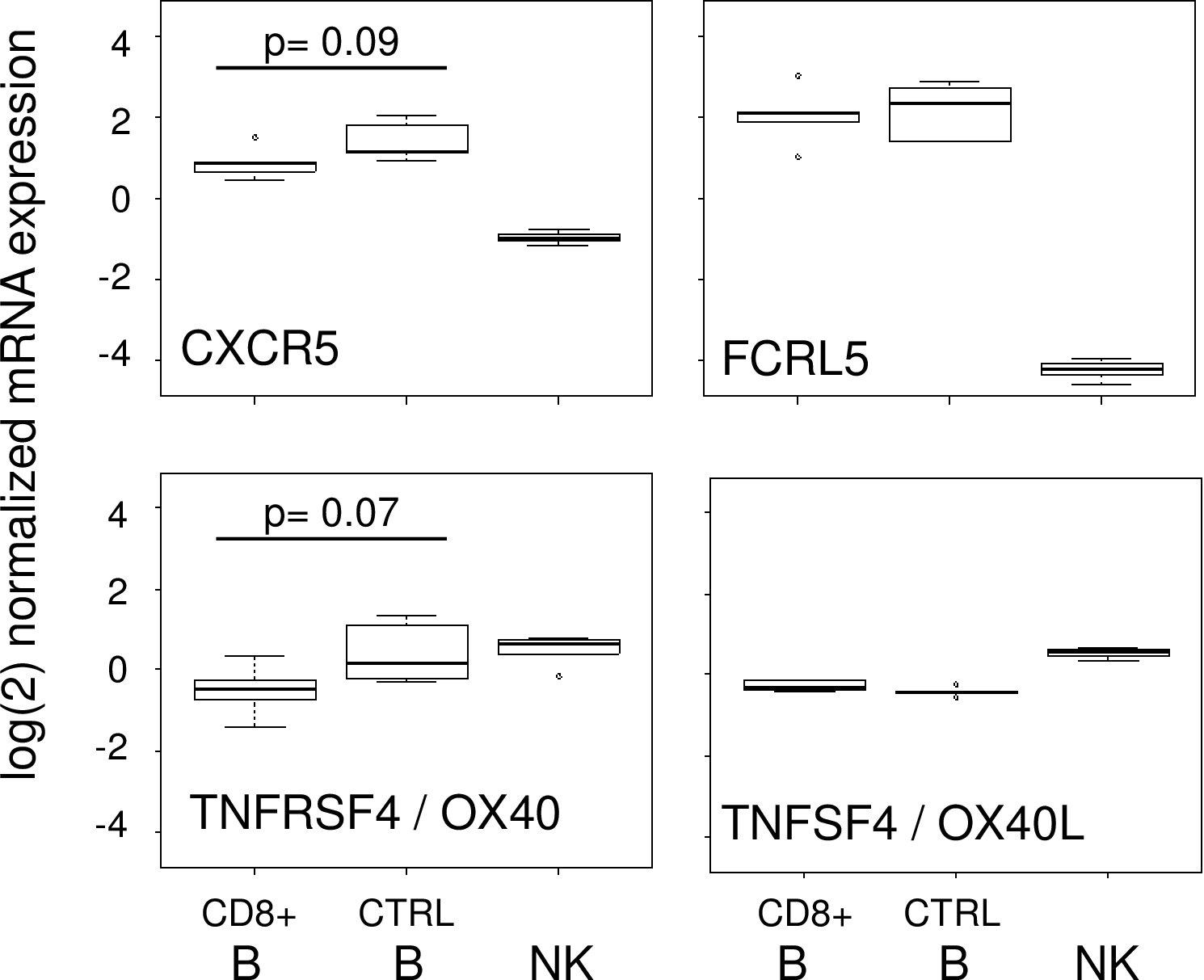

Supplement: S1 Fig — Comparison of genes expressed by CD8+ B cells, control (CTRL) CD8- B cells, and NK cells. Boxplots represent log(2) normalized data obtained from Affymetrix gene chip measurements of cells sorted from five independent apheresis donors. (TIF) [file pone.0208187.s001.tif]

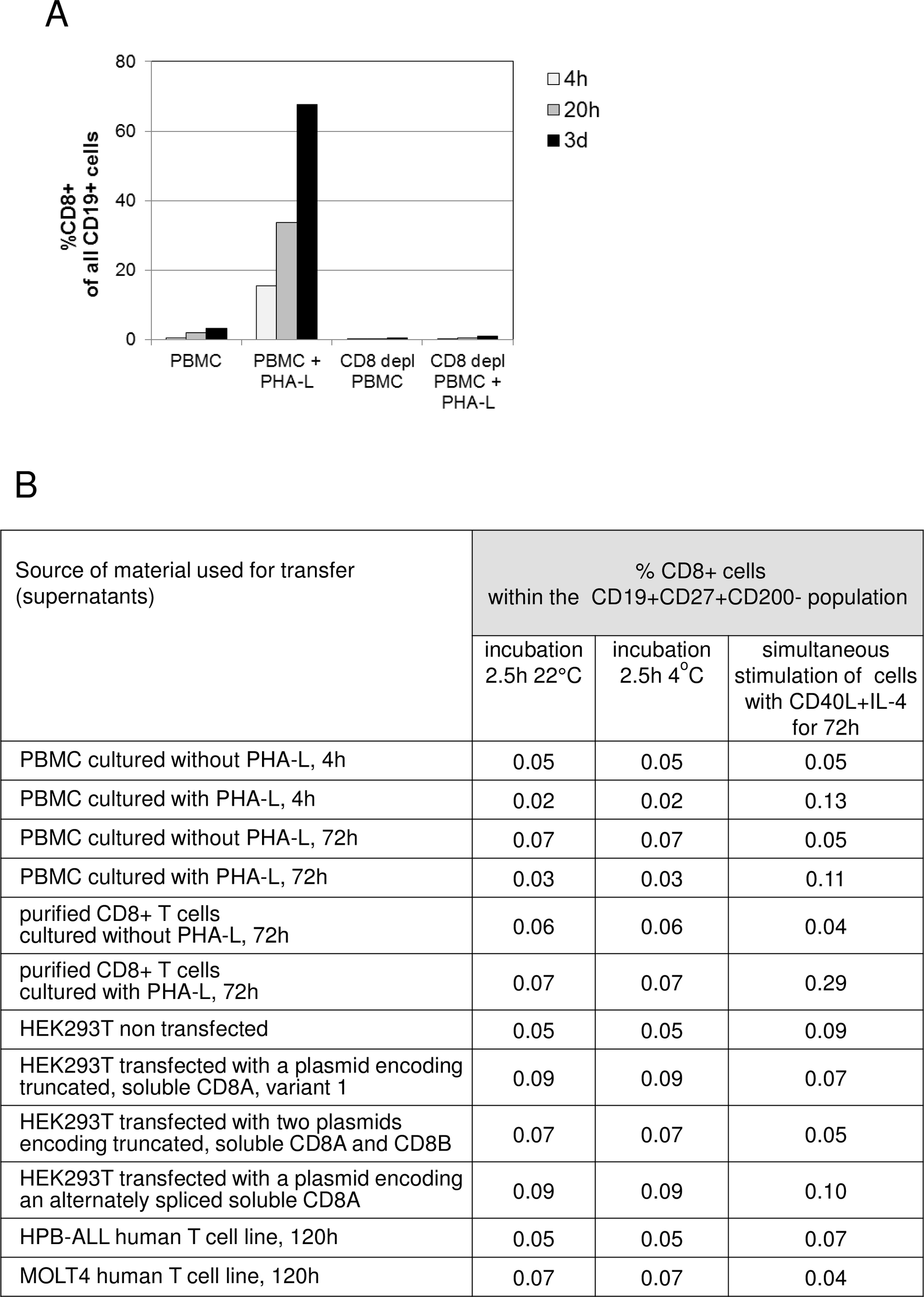

Supplement: S2 Fig — (A) PBMC and CD8 depleted PBMC were cultured with or without PHA-L stimulation (1 μg/ml) for the indicated time. (B) Summary of other transfer tests. Supernatants were obtained from various cell culture preparations, and incubated with purified B cells. Results are expressed as percent of CD8+ cells within the CD19+CD27+CD200- population. (TIF) [file pone.0208187.s002.tif]

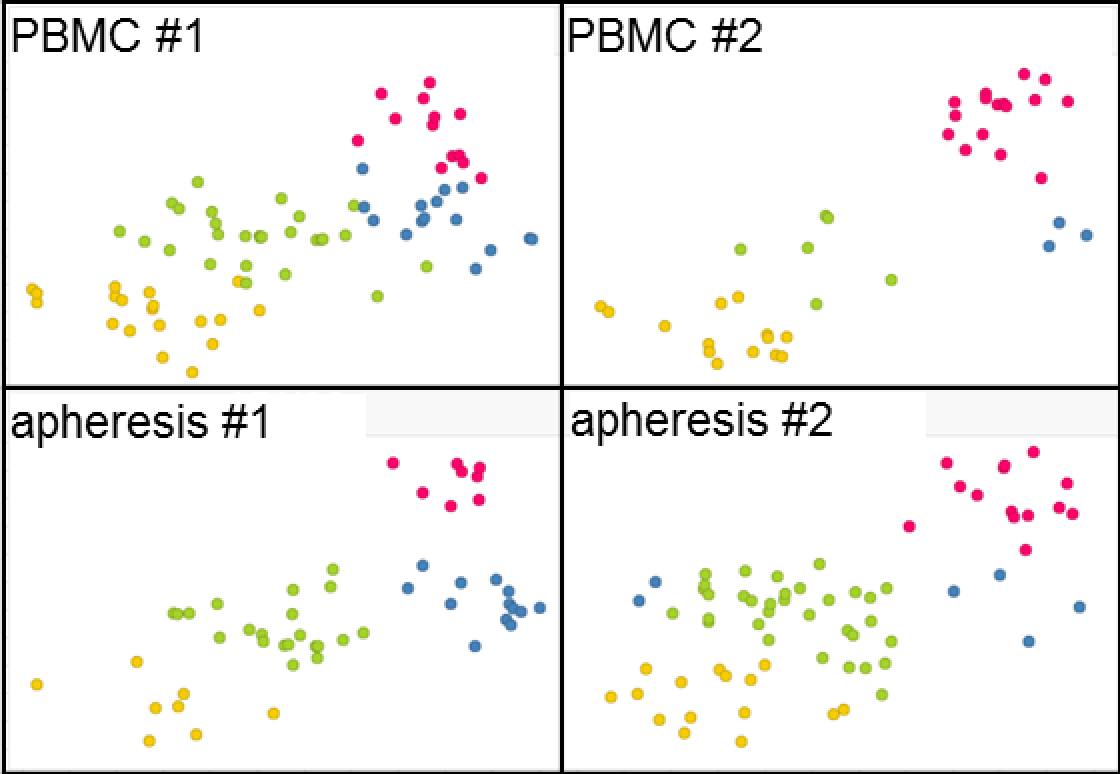

Supplement: S3 Fig — Two-dimensional t-SNE plot of single cells obtained from two PBMC (upper panels) and two apheresis (lower panels) donors. (TIF) [file pone.0208187.s003.tif]

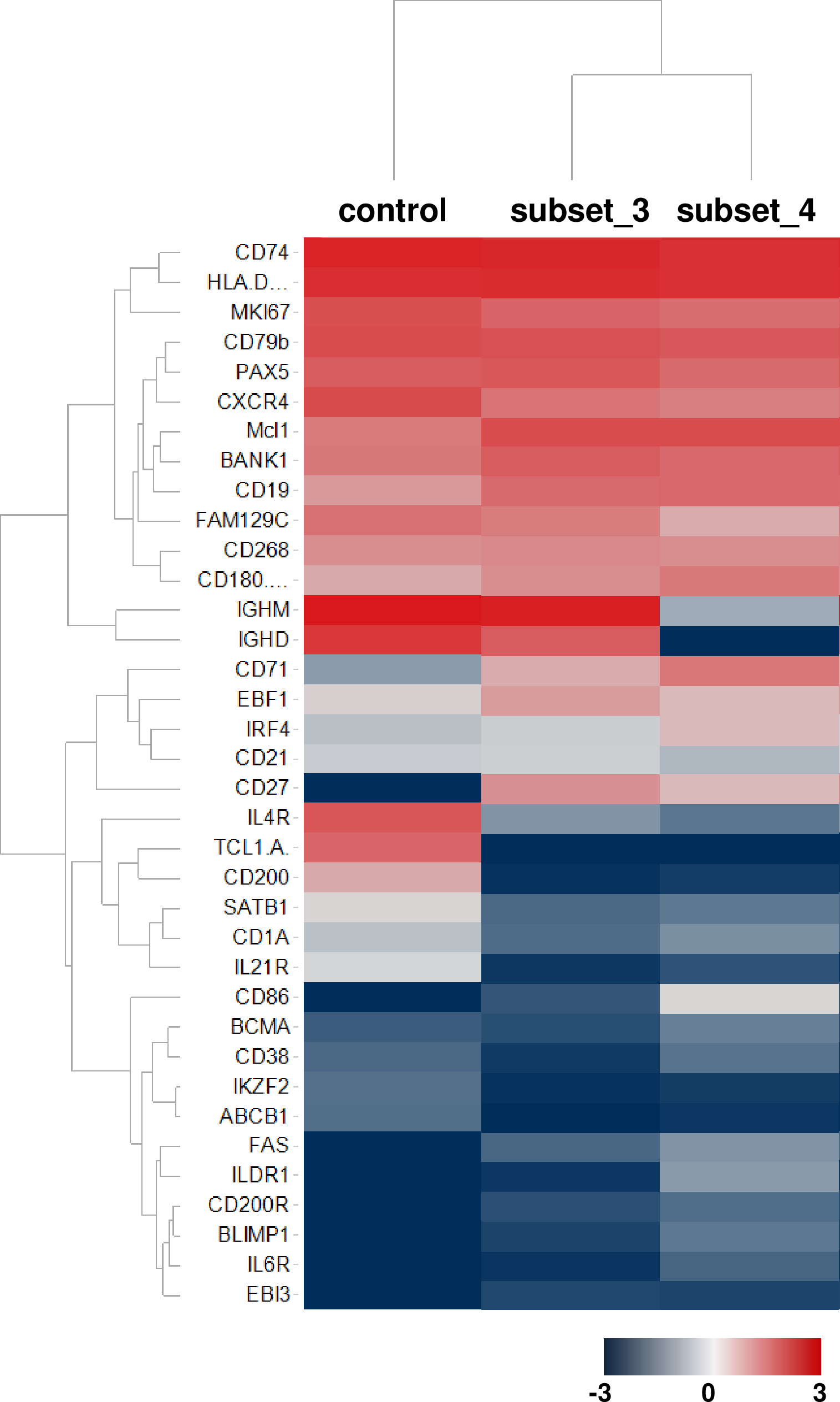

Supplement: S4 Fig — Comparison of control B cells and subsets 3 and 4, based on the converted relative log expression of all tested genes. (TIF) [file pone.0208187.s004.tif]
